# Salinity Is a Key Determinant for the Microeukaryotic Community in Lake Ecosystems of the Inner Mongolia Plateau, China

**DOI:** 10.3389/fmicb.2022.841686

**Published:** 2022-04-12

**Authors:** Changqing Liu, Fan Wu, Xingyu Jiang, Yang Hu, Keqiang Shao, Xiangming Tang, Boqiang Qin, Guang Gao

**Affiliations:** ^1^State Key Laboratory of Lake Science and Environment, Nanjing Institute of Geography and Limnology, Chinese Academy of Sciences (CAS), Nanjing, China; ^2^University of Chinese Academy of Sciences, Beijing, China

**Keywords:** microeukaryotic community, Inner Mongolia Plateau, interspecies interaction, assembly processes, salinity, climate change, lake ecosystem

## Abstract

The arid and semiarid areas experienced remarkable lake shrinkage during recent decades due to intensive human activities and climate change, which would result in unprecedented changes of microeukaryotic communities. However, little is known about how climate change affects the structure and ecological mechanisms of microeukaryotic communities in this area. Here, we used an 18S rRNA gene-based high-throughput sequencing approach to explore the structure, interspecies interaction, and assembly processes of the microeukaryotic community in lake ecosystems of the Inner Mongolia Plateau. As a direct result of climate change, salinity has become the key determinant of the lacustrine microeukaryotic community in this region. The microeukaryotic community in this ecosystem can be divided into three groups: salt (Lake Daihai), brackish (Lake Dalinuoer) and freshwater lakes. Co-occurrence network analysis revealed that salinity shapes the interspecies interactions of the microeukaryotic community. This causes interspecies interactions to change from antagonistic to cooperative with an increase in salinity. Phylogenetic-based β-nearest taxon distance analyses revealed that stochastic processes mainly dominated the microeukaryotic community assembly in lake ecosystems of the Inner Mongolia Plateau, and salinity stress drove the assembly processes of the microeukaryotic community from stochastic to deterministic. Overall, these findings expand the current understanding of interspecies interactions and assembly processes of microeukaryotic communities during climate change in lake ecosystems of the Inner Mongolia Plateau.

## Introduction

Global climate change is expected to directly and indirectly alter the community structure and ecosystem functioning of lakes worldwide (Woodward et al., 2010; Bellard et al., 2012), which will lead to changes in mean ambient temperature and precipitation patterns worldwide (Knutti and Sedlacek, 2013). Arid and semiarid areas display the most significant increases in temperature over the past 100 years (Ji et al., 2014), and climate change would enhance drought conditions with strong implications for the water level and salinity in lakes (Trenberth et al., 2014). Therefore, salinity would be a key determinant of the community structure and ecosystem functioning of lakes in arid and semiarid areas worldwide during the climate change.

Salinity has a significant effect on the microbial community composition and diversity of lakes (Williams, 1998; Zhong et al., 2016), as well as to changes in microbial interactions (Ji et al., 2019). However, previous studies have generally been limited to prokaryotic microorganisms (Tang et al., 2012; Zhong et al., 2016; Banda et al., 2020) while paying less attention to microeukaryotes (Wu et al., 2009; Li et al., 2021). As important components of food webs (McCarthy et al., 2007), planktonic microeukaryotes play a range of ecological roles in lake systems, such as primary producers (Callieri, 2008; Wilken et al., 2018), bacterivores (Boenigk and Arndt, 2002), parasites (Grossart et al., 2019), and saprotrophs (Wurzbacher et al., 2010). Thus, their response to salinity fluctuations may directly influence the structure and function of lake ecosystems in arid and semiarid areas.

Changes along salinity gradients would not only drive the community composition but also the ecological interactions of microeukaryote (Mo et al., 2021). Those complicated ecological relationships of microeukaryotic communities could be represented as co-occurrence networks, which is fundamental for characterizing species interactions and dynamics of lake ecosystems (Berry and Widder, 2014). Although co-occurrence networks may not always reflect true ecological relationships (Freilich et al., 2018), it could help understand the interspecies interactions of microeukaryotes and how such interspecies interactions might change in response to salinity and how interspecies interactions might have implications for ecosystem functioning.

Deterministic and stochastic processes simultaneously affect the assembly of microbial communities (Wang et al., 2013; Dini-Andreote et al., 2015; Logares et al., 2018), and the structure of microeukaryotic communities can be assumed to depend on the balance between stochastic and deterministic processes (Hou et al., 2020). Unraveling the mechanisms of microbial community assembly across environmental gradients, such as lake trophic gradients (Zeng et al., 2019) and bloom stages (Xue et al., 2018), is a central facet of lake microbial ecology. To date, few studies have focused on microeukaryotic community assembly processes, especially across salinity gradients in lakes.

The Inner Mongolia Plateau, where lakes are widely distributed, is a typical arid and semiarid zone in China. However, the Inner Mongolia Plateau has experienced remarkable lake shrinkage during recent decades due to intensive human activities and climate change (Tao et al., 2015). These drastic changes in lakes have led to salinity changes, which would be a key determinant of co-occurrence patterns and assembly processes of microeukaryotic communities in the Inner Mongolia Plateau. Here, for the first time, we investigated the structure, interspecies interaction and assembly processes of microeukaryotic communities using 18S rRNA gene-based high-throughput sequencing in the lake ecosystem of the Inner Mongolia Plateau. The purpose of this study was to (1) determine how the diversity and composition of the microeukaryotic community varies along natural salinity gradients in lake ecosystems, (2) identify whether salinity affects interspecies interactions of microeukaryotic communities along natural salinity gradients, and (3) explore how salinity affects microeukaryotic community assembly processes.

## Materials and Methods

### Sampling and Environmental Information

The sampling sites were along an east-to-west transect in the Inner Mongolia area, China, at 40.44°N to 43.45°N and 112.27°E to 116.91°E (Supplementary Figure 1). In this study, 41 surface water samples (50 cm depth) of lakes were collected in September 2018 from Lake Daihai, Lake Dalinuoer, Lake Durenaoer, Lake Chagannaoer, Lake Ganggengnuoer and inflow rivers (Supplementary Table 1). Approximately 300 mL of lake water was filtered through 0.22 μm polycarbonate filters (47 mm diameter, Millipore, Billerica, MA, United States) to collect microeukaryotes. Filters were stored at –80°C until further processing. In total, 20 environmental variables in the water were measured.

Physicochemical parameters include water temperature (WT), conductivity (Cond), total dissolved solids (TDS), salinity, pH, turbidity (NTU), dissolved oxygen (DO) and fluoride-dissolved organic matter (Fdom), were recorded onboard with a multiparameter water quality sonde (YSI 6600 v2, Yellow Springs Instruments Inc., United States). The concentrations of total nitrogen (TN) and total dissolved nitrogen (TDN) were determined through colorimetry after digestion. Nitrate (NO_3_^–^), ammonium (NH_4_^+^), total phosphorus (TP), total dissolved phosphorus (TDP) and phosphate (PO_4_^3–^) were measured using a continuous flow analyzer (San Plus system, Skalar, Breda, The Netherlands) following the manufacturer’s instructions. Chlorophyll a (Chl-a) was measured using the acetone method after extraction overnight in 90% acetone. The chemical oxygen demand (COD) was analyzed according to the alkaline potassium permanganate method. Suspended solids (SS), loss on ignition (LOI), and inorganic suspension solids (ISS) were determined in the laboratory according to standard methods.

The estimation of the *TLI* is described in detail in Wang (2002). The MAP and MAT were extracted from the Climatic Research Unit (CRU) Time-Series (TS) version 4.05 (Harris et al., 2020). The lake area in the mid-1980s and 2015 was acquired from Tao et al. (2020).

### DNA Extraction and Sequencing

DNA was extracted using the FastDNA^®^ Spin Kit for Soil (MP Biomedicals) according to the manufacturer’s instructions. The 18S rRNA genes were amplified by polymerase chain reaction (PCR) using the universal eukaryote primers Ek-NSF573 (5′-CGCGGTAATTCCAGCTCCA-3′) and Ek-NSR951 (5′-TTGGYRAATGCTTTCGC-3′) targeting the V4 region of most aquatic microeukaryote 18S rRNA genes (Mangot et al., 2013). PCR amplification was performed using a touchdown program as previously described (Liu et al., 2020). The amplicons were then sent for sequencing on an Illumina HiSeq platform at the Beijing Genomics Institute (Shenzhen, China). Sequences have been deposited at NCBI under BioProject numbers PRJNA755855.

After filtering raw reads by removing adaptors and low-quality and ambiguous bases, paired-end reads were added to tags by FLASH (v1.2.11) (Magoc and Salzberg, 2011) to obtain the tags. The tags were clustered into OTUs with a 97% similarity threshold using UPARSE (v7.0.1090) (Edgar, 2013), and chimera sequences were compared with the Gold database using UCHIME (v4.2.40) (Edgar et al., 2011) for detection. Representative sequences of each OTU were taxonomically classified using an 80% confidence threshold against the SILVA 132 database (BLAST threshold *e*-value = e^–6^) for taxonomic annotation. To prevent artificial diversity inflation, OTUs appearing in only one sample were removed. OTUs identified as multicellular animals (Metazoa) and plants (Streptophyta) were removed.

### Statistical Analyses

The statistical analyses were performed using R 4.1.0^1^, unless otherwise indicated. Canonical correlation analysis (CCA) was also used to test the correlation between environmental variables and microeukaryotic community structure. Variance inflation factors (VIFs) were used to identify the autocorrelated factors, and environmental variables with VIF values < 10 were selected for CCA. CCA was conducted using the *cca* function from the ‘vegan’ R package, and VIFs were calculated using *vif cca* function in the ‘vegan’ R package (Dixon, 2003). All environmental variables that showed a significant relationship with the microeukaryotic community in CCA were selected to perform variation partitioning analysis (VPA), which was used to quantify the effect of environmental factors on microeukaryotic community variation. A partial Mantel test was used to estimate the effect of salinity distance on microeukaryotic community structure after controlling for spatial distance and other environmental distances (Borcard et al., 2011), excluding salinity. Pairwise geographic distances between samples were calculated from latitude and longitude coordinates using the “geosphere” package in R (Karney, 2013), and beta diversity was measured using Bray-Curtis dissimilarity. Partial Mantel tests were conducted using the *mantel partial* function from the ‘vegan’ R package.

MRT analysis was also performed to detect relationships between microeukaryotic community structure and environmental variables (De’Ath, 2002). A total of 1,000 cross-validations using the “lse” method were used to decrease the complexity of the tree to identify the main predictors of microbial community structure. MRT analysis was conducted using the “mvpart” package in R. Microeukaryotic community composition was visualized using non-metric multidimensional scaling (NMDS) based on Bray-Curtis dissimilarities. The differences of microeukaryotic communities in different salinity gradients were evaluated by permutational multivariate analysis of variance (PERMANOVA) using *adonis* function in the ‘vegan’ R package based on Bray–Curtis dissimilarity (Clarke, 1993). We determined which OTUs could explain the salinity effect in the microeukaryotic community by identifying indicator species in the “indicspecies” R package (De Caceres and Legendre, 2009). We compared samples across three salinity gradients (salt lakes, brackish lakes and freshwater lakes) to determine salinity indicators based on an indicator value > 0.6 and *p value* < 0.001 assessed after 999 permutation tests.

In an attempt to acquire the best discriminant performance of taxa in different salinity gradients in lake ecosystems of the Inner Mongolia Plateau, we regressed the relative abundances of microeukaryotic taxa at the order level against salinity gradients in lake ecosystems using the 10-fold cross-validation of the *rfcv* function in the “randomForest” package in R (ntree = 1000) with five repeats (Zhang et al., 2018). The order number against the cross-validation error curve stabilized and reached the minimum value when using 20 important orders; therefore, the 20 most important orders were chosen as marker taxa correlated with salinity gradients in the lake ecosystem of the Inner Mongolia Plateau (Edwards et al., 2018). The Wilcoxon test was used to test the difference between the actual values and the predicted values, which was calculated from random forest (RF) model using the *wilcox test* function in “stats” R package. To further estimate the contributions of individual environmental variables in microeukaryotic communities, we also regressed the environmental variables against alpha diversity using the “randomForest” package in R (ntree = 1000). The community composition was represented by the MDS1 of NMDS based on Bray-Curtis distance.

### Network Analysis

To explore the co-occurrence patterns of the microeukaryotic community at different salinity gradients, three co-occurrence networks were constructed based on Spearman’s rank correlation. To reduce the complexity of the datasets, only OTUs that occurred in at least 25% of the samples were selected to construct co-occurrence networks. All possible pairwise Spearman’s rank correlations (ρ) between those OTUs were calculated with the “psych” R package. Only robust (|ρ| > 0.6) and statistically significant (FDR-adjusted *p value* < 0.05) correlations were incorporated into network analyses. Topological properties (i.e., degree, betweenness centrality, clustering coefficient, average path length, modularity and network diameter) were further calculated in the “igraph” R package. Meanwhile, 10000 Erdős-Rényi random networks were generated in the “igraph” R package, which had identical scales (the identical number of nodes and edges) as the real co-occurrence network, with each edge having the same probability of being assigned to any node (Lieberman et al., 2005). The topological properties of the random network were also calculated and compared with those of real networks. To identify the proportional influence of various microeukaryotic taxa in a network structure along natural salinity gradients, we calculated the network degree proportion of order (Strogatz, 2001). To further assess the effects of environmental variables on microeukaryotes, we also constructed co-occurrences between microeukaryotes and environmental variables. Network visualization and modular analysis were performed with Gephi version 0.9.2.

### Microeukaryotic Community Assembly Processes

To infer microeukaryotic community assembly processes, the phylogenetic turnover between communities among different salinity gradients was quantified using the β-nearest taxon index (βNTI). βNTI measures the deviation of observed β-mean nearest taxon distance (βMNTD) from mean βMNTD in the null model, in which taxa are randomized across the tips of phylogenetic trees. In addition, βNTI combined with Bray-Curtis-based Raup-Crick (RC_Bray_) was then applied to quantify the relative contributions of ecological assembly processes (Chase et al., 2011; Stegen et al., 2013, 2015). βNTI was calculated in the R ‘picante’ package (Kembel et al., 2010), and RCbray were calculated by the determination of the deviation between the empirically observed Bray-Curtis data and the null distribution using “vegan” package. When | βNTI| > 2, microeukaryotic community assembly was dominated by deterministic processes, including variable selection (βNTI > 2) and homogeneous selection (βNTI < −2). Whereas | βNTI| values below 2 indicated the dominance of stochastic processes, including homogenizing dispersal (RC_bray_ < −0.95), dispersal limitation (RC_bray_ > 0.95) and undominated (−0.95 < RC_bray_ < 0.95) (Dini-Andreote et al., 2015).

## Results

### Environmental Characteristics

The physicochemical properties of water samples from lakes are summarized in Supplementary Figure 2. The lakes were characterized as salt (Lake Daihai; salinity, ∼11.3‰), brackish (Lake Dalinuoer; salinity, ∼6.2‰) and freshwater (other lakes and inflow rivers; salinity, < 1.0‰). Lake eutrophication is common in lakes of the Inner Mongolia Plateau, and the trophic level index (*TLI*) of all the lakes is higher than 60 (Supplementary Figure 3). For the last forty years, the mean annual temperature (MAT) of all sampling lakes increased significantly (*p* < 0.0001) (Supplementary Figure 4), although no significant changes in mean annual precipitation (MAP) were observed (Supplementary Figure 5). As temperatures rose, shrinkage was observed in most lakes, and Lake Chagannaoer dried up in 2015 (Supplementary Figure 6).

### Microeukaryotic Community Compositions

The sequencing of 18S rRNA genes yielded 1030100 high-quality sequences and 2,577 OTUs at a 97% similarity level (Supplementary Table 2). These sequences were assigned to three microeukaryotic groups, algae (601050 sequences, 570 OTUs), protozoa (283864 sequences, 1113 OTUs) and fungi (130166 sequences, 686 OTUs). The non-pigmented taxa (protozoa and fungi) dominated the OTU richness, together representing 69.81% of the total OTUs. Pigmented groups (algae) dominated the OTU abundance, accounting for 58.35% of microeukaryotic sequences. The dominant microeukaryotes of different taxa at the phylum level were Chlorophyta (average relative abundance, 30.90%), Ciliophora (8.87%), and Cryptomycota (4.90%).

The 10 most abundant OTUs were mainly affiliated with algae, except OTU16 and OTU15, and showed variable distribution patterns in different lakes. OTU13 (*Tetraselmis* sp.), OTU3 (*Peridinium* sp.), OTU8 (*Scrippsiella* sp.), and OTU1826 (unclassified Oocystaceae) were all most abundant in Lake Daihai. OTU9 (*Marvania* sp.), OTU5 (*Nannochloris* sp.), OTU16 (*Perkinsidae* sp.), OTU49 (*Ankyra judayi*) and OTU15 (*Fragilaria* sp.) were highly abundant in the Lake Dalinuoer. In addition, OTU7 (*Fragilaria* sp.) was the only abundant OTU in some freshwater lakes (Supplementary Figure 7).

### Driving Factors and Patterns of Microeukaryotic Community Structure

The CCA results showed that salinity was the most important factor that determined microeukaryotic community structure of the Inner Mongolia Plateau and explained 11.62% of its total variation (Table 1). The VPA results further indicated that salinity, TP and other environmental variables explained 10.68, 10.14, and 43.99% of the observed variation, respectively, leaving 42.16% of the variation unexplained (Supplementary Figure 8).

**TABLE 1:** Microeukaryotic community of variance explained by environmental variables according to CCA.

Predictor variables	Explained variance	*F*	*P*
Salinity	11.62%	9.0797	<0.001
TP	10.16%	7.9393	<0.001
Chla	8.12%	6.3457	<0.001
NO_3_^–^	7.01%	5.4779	<0.001
Fdom	6.61%	5.1619	<0.001
NH_4_^+^	6.06%	4.7352	<0.001
ISS	5.69%	4.442	<0.001
WT	3.75%	2.9322	<0.001

**FIGURE 1:**
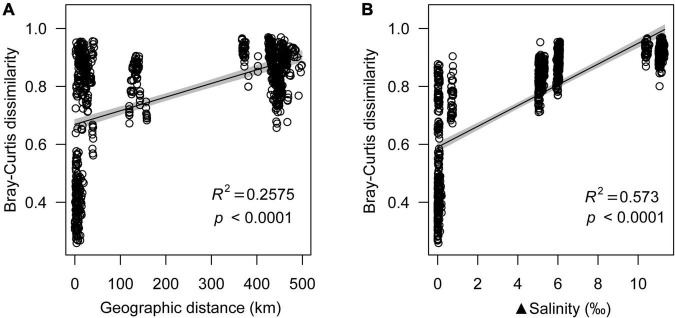
Relationship between geographic distance **(A)** and salinity **(B)** with Bray-Curtis dissimilarity.

Geographic distance was also an important factor of microeukaryotic community variation, which showed a significant positive relationship with microeukaryotic Bray-Curtis dissimilarity (R^2^ = 0.2575, *p* < 0.0001) (Figure 1A). Even though both salinity and geographic distance had significant effects on the microeukaryotic community structure (*p* < 0.0001), the effect of salinity (R^2^ = 0.4702) was stronger than that of geographic distance (R^2^ = 0.2968) (Supplementary Table 3).

**FIGURE 2:**
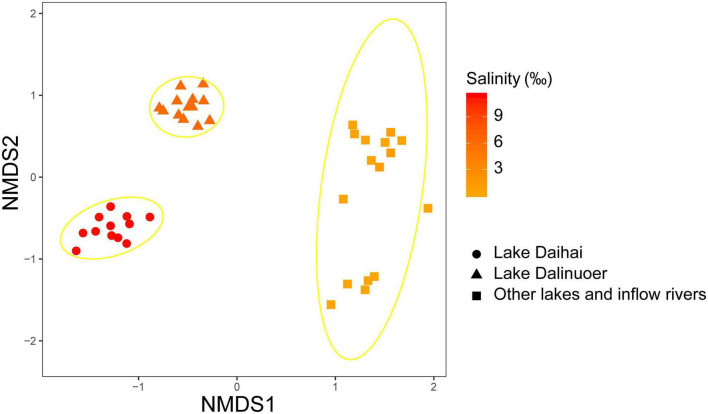
NMDS analysis based on Bray-Curtis dissimilarity with 95% confidence ellipses represented for each salinity level. The shape and color of the point represents the group and salinity of sample.

Microeukaryotic Bray-Curtis dissimilarity showed a significant positive relationship with differences in salinity (R^2^ = 0.573; *p* < 0.0001), and microeukaryotic community dissimilarity increased with increasing salinity difference (Figure 1B). NMDS analyses revealed that the microeukaryotic community could be divided into three groups based on salinity (Figure 2), including salt (Lake Daihai), brackish (Lake Dalinuoer) and freshwater lakes (other lakes and inflow rivers). The PERMANOVA results also showed that the microeukaryotic communities under the three salinity gradients were significantly different (*p* < 0.001). The MRT result was consistent with the NMDS result, which also divided the data into three groups based on salinity (Supplementary Figure 9), including salt (≥ 8.75 ‰), brackish (≥ 3.535 ‰ and < 8.75 ‰) and freshwater lakes (< 3.535 ‰).

### The Response of Microeukaryotes in Lake Ecosystems to Salinity

**FIGURE 3:**
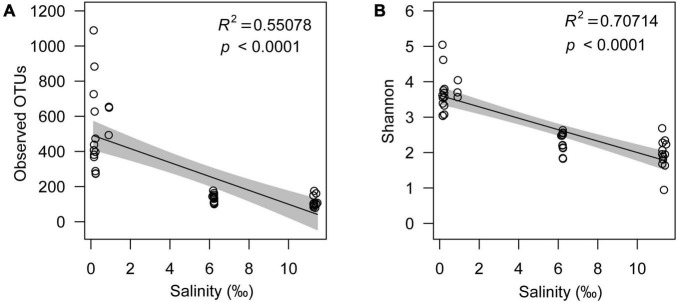
Relationship between salinity and observed OTUs **(A)** and the Shannon index **(B)**.

**FIGURE 4:**
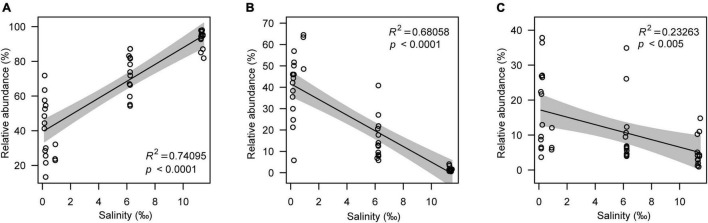
The relative abundance of the main microeukaryotic taxa. **(A–C)** represent algae, protozoa and fungi.

Although the microeukaryotic alpha diversity, including observed OTUs (R^2^ = 0.55078, *p* < 0.0001) and the Shannon index (R^2^ = 0.70714, *p* < 0.0001), decreased with increasing salinity (Figures 3A,B), the response of different microeukaryotes to salinity varied. The relative abundance of algae had a strong positive linear relationship with salinity (R^2^ = 0.74095, *p* < 0.0001); however, protozoa (R^2^ = 0.68058, *p* < 0.005) and fungi (R^2^ = 0.23263, *p* < 0.0001) decreased with increasing salinity (Figures 4A,C). In the algal taxa, although the relative abundances of Chlorophyta and Dinophyceae increased with increasing salinity, the opposite result was observed for Bacillariophyta and Chrysophyceae (Supplementary Figures 10A-D). In the non-pigmented taxa, the relative abundance of most protozoan taxa had a strong positive linear relationship with salinity except Perkinsozoa; however, most fungal taxa had no significant relationship with salinity (*p* > 0.005), except Cryptomycota (Supplementary Figures 10E-l).

These different responses of microeukaryotes to salinity also led to the replacement of dominant groups (Supplementary Figure 11). Algae, the pigmented taxa, were highly diverse and not a dominant group in the freshwater lakes. However, Bacillariophyta, Chlorophyta and Dinophyceae successively become dominant taxa with increasing salinity. There was also a similar pattern in the protozoan taxa, although the dominance of Perkinsozoa only occurred in the brackish lake. As the fungal taxon, Chytridiomycota, lost dominance and was replaced by Ascomycota in the salt lake.

There was high variability in the number of indicator OTUs over the salinity gradient, ranging from 46 in the salt lake, to 71 in the brackish lake, and 91 in the freshwater lakes (Supplementary Figure 12). The taxonomic compositions of indicator OTUs were significantly different among different salinity gradients, and the indicator OTUs with close phylogenetic affiliations tended to co-occur. Almost all OTUs related to Chaetocerotales, Chlorosarcinales, Peridiniales, and Ascomycota were indicators of the salt lake, while almost all OTUs affiliated with Pyrenomonadales, Jakobida, Trebouxiophyceae, Cercozoa, Choanozoa and Chytridiomycota were indicators of the brackish lake, and almost all OTUs belonging to Sphaeropleales, Chromulinales, Saprolegniales, Vampyrellida, Saccharomycetales, Ciliophora and Oomycota were indicators of freshwater lakes.

### Random Forest Model to Correlate Microeukaryotic Biomarkers With Salinity Gradients in Lake Ecosystems

**FIGURE 5:**
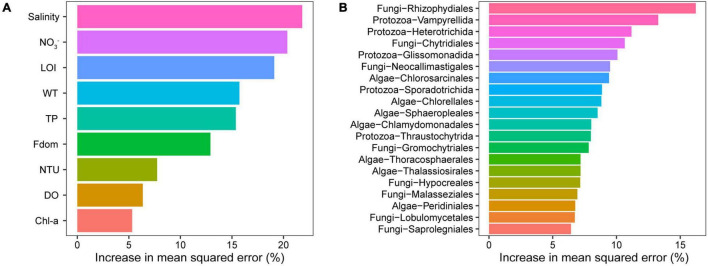
Result of the RF model. **(A)** The relative importance of environmental variables to the microeukaryotic community. **(B)** Microeukaryotic biomarkers (order level) with salinity gradients in lake ecosystems.

The composition (MDS1) of microeukaryotic communities was sensitive to ambient environment especially salinity based on the results of the RF model (Figure 5A). Furthermore, the RF model was constructed using the microeukaryote at the order level, which showed the predictive accuracy of 92.24% for salinity. When the order number exceeded 20, the error rates no longer decreased. Those 20 orders (biomarkers) were identified and were used for the reconstruction of RF model (Figure 5B). The predictive accuracy of the new model was 94.89%, which was slightly higher than that of the model constructed using all microeukaryotic orders. No significant difference (*p* > 0.05) was observed between the actual values and the predicted values of lake salinity, which implied that the predictions are reliable.

### Co-occurrence Patterns of the Microeukaryotic Community

**FIGURE 6:**
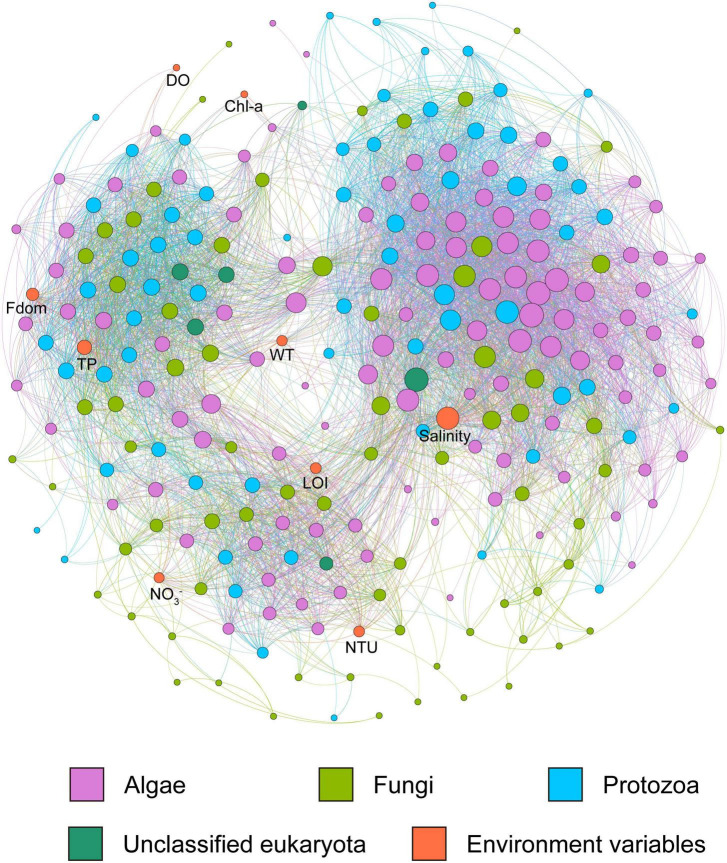
Co-occurrence networks of microeukaryotic community and environmental variables in the lake system of the Inner Mongolia Plateau. The size of the circles shows the degree of the node, and the color of the circles shows the taxon of the node.

In the lake ecosystem of the Inner Mongolia Plateau, salinity, TP, Fdom, NTU, WT, LOI, NO_3_^–^, Chl-a and DO were identified in the microeukaryotic networks. Salinity recorded the highest node connectivity (node degree = 97), which was a key driver of network connections (Figure 6).

The co-occurrence networks for the salt and brackish lakes had scale-free degree characteristics (power law: R^2^ = 0.66 for the salt lake network and R^2^ = 0.88 for the brackish lake network), which were different from their relevant random networks (Erdős–Rényi model), indicating that both real co-occurrence networks were non-random. The modularity, average clustering coefficient and average path length of three real co-occurrence networks were all greater than those of their respective Erdős-Réyni random networks, suggesting that both networks had “small-world” properties and modular structures (Table 2 and Supplementary Table 4). The average connectivity (average degree, avgK) of real co-occurrence networks decreased with increasing salinity, but the average clustering coefficient (avgCC) exhibited the opposite trend. With the decline in salinity, the node numbers and link numbers of real co-occurrence networks would increase. However, the proportion of positive interactions in the co-occurrence networks increased with salinity stress (Table 2 and Supplementary Figure 13).

**TABLE 2:** Topological properties of the microbial network.

Network indices	Salt lake	Brackish lake	Freshwater lakes
Total nodes	92	120	308
Total links	300	554	3,685
R^2^ of the power-law	0.66	0.88	0.03
Average degree (avgK)	6.52173913	9.233333333	23.92857143
Average clustering coefficient (avgCC)	0.390433815	0.506935271	0.551597397
Average path distance (GD)	3.451135494	3.4650927	2.757096324
Modularity	0.4745959	0.3214333	0.4970727
Connectance	0.071667463	0.077591036	0.077943229
Centralization betweenness	0.184856612	0.135467425	0.031203392
Centralization degree	0.126134735	0.216526611	0.153327129
Postive correlation	300	552	3,357
Negative correlation	0	2	328

For the co-occurrence network of the salt lake, Chlorosarcinales (9.78%), Chaetocerotales (4.35%), Chlorellales (4.35%), Pleosporales (3.26%) and Peridiniales (3.26%) mainly occupied the nodes. Nodes in the brackish lake mainly belonged to Pyrenomonadales (4.17%), Chromulinales (4.17%), Chlamydomonadales (4.17%), and Jakobida (3.33%). Chromulinales (7.47%), Chlamydomonadales (4.22%), Perkinsida (4.22%), Sphaeropleales (3.57%) and Vampyrellida (2.92%) mainly occupied the nodes in the freshwater lakes (Supplementary Figure 13). In addition, the nodes from the same taxon co-occurred more frequently in the same modules within themselves than with nodes in other modules. For example, most Pyrenomonadales nodes co-occurred in module I of the brackish lake network.

The lowest proportional influence (network degree proportion) of the algae was observed in the freshwater lakes, while the fungi and protozoa were observed in the brackish and salt lakes, respectively (Supplementary Figure 14A). Various orders exhibited considerable differences in their proportional influence on the complexity of microeukaryotes, and the proportional influence of the major orders changed with salinity variation (Supplementary Figure 14B). Perkinsida, Chromulinales, and Vampyrellida are major orders that influence the freshwater lake networks, while Pyrenomonadales, Chromulinales and Pedinellales showed a major influence in the brackish lake network. In addition, Chaetophorales, Chlorosarcinales and Chlorellales showed a major influence in the salt lake network. Some orders, such as Bicosoecida, Chaetophorales and Chlorellales, have proportional influences that increase with increasing salinity. However, the proportional influences of Perkinsida, Pythiales, Sphaeropleales and Vampyrellida decrease with increasing salinity.

### Assembly Processes of the Microeukaryotic Community

**FIGURE 7:**
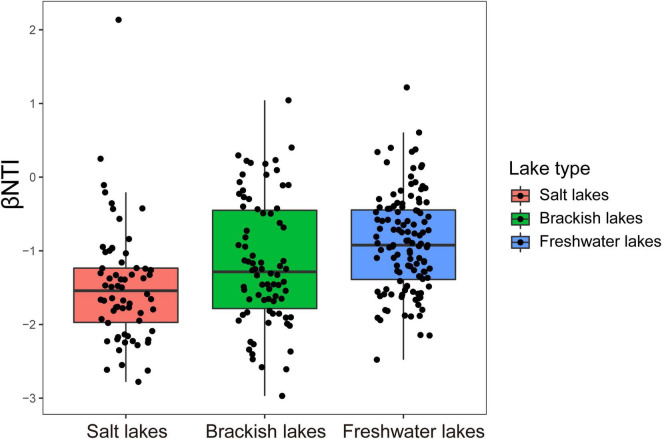
Distribution patterns of βNTI values among lakes with different salinity gradients on the Inner Mongolia Plateau.

βNTI mostly has scores between −2 and + 2, and its distribution progressively shifted with salinity gradients, with values that were negatively correlated with differences in salinity (R^2^ = 0.021091, *p* < 0.0001) (Supplementary Figure 15). βNTI values among the different salinity lakes including salt lake (mean ± SD: −1.55 ± 0.62), brackish lake (mean ± SD: −1.23 ± 0.74) and freshwater lakes (mean ± SD: −0.96 ± 0.56) significantly increased with decreasing salinity (Kruskal-Wallis test, *p* < 0.05) (Figure 7).

βNTI combined with RC_Bray_ was then applied to quantify the relative contributions of ecological assembly processes. The results indicated that deterministic selection (heterogeneous selection and homogeneous selection) (25.75%) and homogenizing dispersal (57.58%) fractions were predominant in shaping the microeukaryotic community in the salt lake. In the brackish lake, homogenizing dispersal (78.21%) played the most important role in the assembly of the microeukaryotic community. However, dispersal limitation (88.33%) was the most influential portion governing the microeukaryotic community in freshwater lakes (Supplementary Figure 16).

## Discussion

### Salinity Governs the Microeukaryotic Community

Salinity is a dominant environmental selective force governing the microbial community in natural inland lakes (Wu et al., 2006; Wang et al., 2011; Zhong et al., 2016), whose importance may be rather extreme compared to other physical and chemical factors (Lozupone and Knight, 2007), such as temperature and pH. In the lake ecosystems of the Inner Mongolia Plateau, salinity was the most important environmental driver of microeukaryotic community structure across the samples from freshwater lakes to the salt lake (Supplementary Figure 8 and Table 1), and salinity stress reduced the diversity of the microeukaryotic community (Figure 3). Similarly, the reduction in diversity of aquatic microorganisms is common when salinity increase, such as lake bacteria (Zhong et al., 2016) and reservoir microeukaryotes (Mo et al., 2021). Most microorganisms become inactive and even die with increasing salinity because of the rise in extracellular osmolarity (Oren, 2011; Rath and Rousk, 2015; Zhang et al., 2019) and generation of reactive oxygen species (Shetty et al., 2019); thus, the diversity of the microeukaryotic community decreases with increasing salinity.

In addition to microeukaryotic diversity, salinity also governed the microeukaryotic structure and was the most important factor that best explained the variances in the microeukaryotic community. Natural ecosystems with different salinity ranges usually are inhabited by distinct microbial communities due to the specific tolerance or requirement of microorganisms (Oren, 2011). Although high salinity levels reduce algal abundance and activities, a slight increase of salinity is often accompanied by relatively high nutrient levels, which would promote algae growth (Yue et al., 2019; Li et al., 2021). In Contrast with protozoa and fungi, the relative abundance of algae increased with increasing salinity (Figure 4A-C), which caused distinct microbial communities at different salinity levels. Furthermore, a rise in temperature or a reduction in rainfall would reduce the lake area and exacerbate lake salinization on the Mongolian Plateau (Wang et al., 2015; Tao et al., 2020); this would increase the salinity stress of the microeukaryotic community.

Apart from climate change (Supplementary Figures 4-6), human activities, such as water conservancy construction and agricultural production, have also resulted in unprecedented changes in lakes (Tao et al., 2015). Human activities exacerbate the shrinkage of lake area, which may cause the salinization of lake and further increase the influence of salinity stress on microeukaryotic communities. In addition, human impacts have also accelerated lake eutrophication in the Inner Mongolia Plateau, which was observed in most lakes sampled in this study. Our research also reveals that the trophic status (TP) of the lake became the secondary driving force of microeukaryotic community succession, whose stress would increase with an economic boom.

### Biomarker Taxa Correlated With Salinity in the Lake

Similar to other lakes (Wang et al., 2014; Filker et al., 2016), pigmented taxa of microeukaryotes were more abundant in lake ecosystems of the Inner Mongolia Plateau, such as Chlorophyta, Dinophyceae and Bacillariophyta. However, the community composition and relative abundance of most microeukaryotic taxa shifted along the salinity gradient, which would be related with physiological mechanisms of different taxa (Rojas-Jimenez et al., 2019). In previous research, microeukaryotes are considered one of the biomarker taxa of lake ecosystems owing to their sensitivity to environmental disturbances (Liu et al., 2015; Xue et al., 2018). On the Inner Mongolia Plateau, microeukaryotes can be considered biomarker taxa of lake salinity owing to their different responses to salinity. For example, Chlorosarcinales were abundant in the salt lake and could be considered a biomarker taxon (Supplementary Figure 12), while the Chlorophyceae and Sphaeropleales orders were indicators of freshwater lakes.

However, the multidimensional and noisy data sets are common in the high-throughput sequencing data. To explore the ecological signal from background noise, machine learning algorithms (such as RF model) have been attempted to prove correlations between high-throughput sequencing data and environmental stressors (Sagova-Mareckova et al., 2021). We identified microeukaryote taxa at the order level that were discriminant of lake salinity using the RF model, therefore it is likely that this model allows for generalization of predictability across continental scales compared with lower-resolution taxonomic levels (Edwards et al., 2018). Using these sets of orders, we were able to accurately predict the lake salinity on the Inner Mongolia Plateau (Figure 5B). Long-term lake datasets would benefit conservation practices and environmental management (Goldewijk et al., 2011; Korosi et al., 2017); however, such datasets are lacking on the Inner Mongolia Plateau. Considering the well environment prediction of microeukaryote in RF model, we even could accurately predict historic salinity fluctuations of the lakes by using sedimentary DNA (Yang et al., 2015; Balint et al., 2018).

### Salinity Shapes Interspecies Interactions of Microeukaryotic Communities

Co-occurrence network analysis offers critical insights into microbial interactions and ecological assembly rules (Zhou et al., 2010; Deng et al., 2012) and has been widely applied in microeukaryotic research (Xue et al., 2018; Hou et al., 2020). We applied correlation-based network analysis to explore the microbial interactions of the microeukaryotic community for the three salinity groups. All networks had “small-world” properties and modular structures (Table 2 and Supplementary Table 4), implying strong interspecies interactions between microeukaryotic communities in the Inner Mongolia Plateau (Watts and Strogatz, 1998).

The topology of the networks, such as average connectivity, average clustering coefficient and modularity, can reflect interactions between microorganisms (Ma et al., 2016; Liu et al., 2020). The average connectivity of real co-occurrence networks decreased with increasing salinity, but the average clustering coefficient exhibited the opposite trend; this indicates that associations and interactions between the microeukaryotic community would weaken with increasing salinity on the Inner Mongolia Plateau (Escalas et al., 2019). Microeukaryotic diversity decreased with increasing salinity (Figure 3), therefore, the diversity reduction would lead to the loss of certain interactions. Consistently, the network complexities of prokaryotic community in lake also decrease with increasing salinity with its value ranging between 0.7 and 35g⋅L^–1^ (Yang et al., 2021). Therefore, co-occurrence networks of microeukaryotic community may have different trend in hypersaline lake, it is possible that our findings may be somewhat limited by the gradients of salinity levels in the sampled lakes.

Although the interaction decreased with increasing salinity, the proportion of positive interactions increased with increasing salinity. A positive interaction in the network indicates the existence of similar niches or mutualisms (Paver et al., 2013), while a negative interaction indicates the potential for a non-overlapping niche or antagonism (Faust and Raes, 2012; Freilich et al., 2018), such as amensalism, prey-predator relationships and competition. Thus, salt stress drives the transformation of interactions in the microeukaryotic community from antagonistic to cooperative on the Inner Mongolia Plateau.

The network of microeukaryotes in lakes with different salinity demonstrated distinct co-occurrence patterns. This implies that not only the response of interspecies interactions to salinity but also implications of interspecies interactions for ecosystem functioning. Every microeukaryotic taxa plays the specific ecological role in lake ecosystem, the response of different taxa to salinity would be connected with ecosystem functioning. Thus, the difference in the major influence order across the microeukaryotic networks may indicate the functional succession of lake ecosystem during salinity changes. For example, the network degree proportion of heterotrophic protozoa (important consumers) (Sherr and Sherr, 2002) in salt lakes was lower than freshwater and brackish lakes, which implied the changes of microbial loop.

### Salinity Drives Assembly Processes of Microeukaryotic Communities Through Stochastic to Deterministic Processes

Deterministic and stochastic processes govern the microbial community and regulate its assembly (Stegen et al., 2012), which is influenced by environmental perturbations (Zhou et al., 2014). In the present study, βNTI mostly had scores between −2 and + 2 (Supplementary Figure 15), implying that stochastic processes dominate the assembly processes of the microeukaryotic community in the Inner Mongolia Plateau (Dini-Andreote et al., 2015). However, we also demonstrated the succession of assembly processes in the microeukaryotic community among different salinity gradients, and the relative importance of deterministic processes improved as salinity increased (Figure 7). This result is consistent with recent findings of prokaryotic community in lakes (Tang et al., 2021; Yang et al., 2021). In our study and previous research (Zhong et al., 2016; Zhang et al., 2019), salinity was a key determinant factor that significantly affected the diversity, composition and interspecies interactions of the microeukaryotic community. Therefore, salinity, serving as a kind of restrictive selection pressure, would increase niche selection (Chase, 2007; Zhou et al., 2014; Zhang et al., 2019); this implies that stronger selective pressure may be exerted on microeukaryotic community when salinity increased. Our results implies that salinity may drive the assembly processes of the microeukaryotic community from stochastic processes to deterministic processes in the Inner Mongolia Plateau. Furthermore, climate change improves salinity fluctuations in the lakes of the Inner Mongolia Plateau, which implies that the relative contributions of deterministic processes of the microeukaryotic community would be stronger with climate change and might select for specific traits (such as salt-tolerant species).

Previous research describes four basic processes (selection, diversification, drift, and dispersal) that contribute to community assembly (Nemergut et al., 2013). In lakes of the Inner Mongolia Plateau, we found that the relative contributions of selection (deterministic processes), especially homogeneous selection, improved with increasing salinity (Supplementary Figure 16). The homogeneous selection process means that environments constrain the divergence of microbial populations (Zhou and Ning, 2017). The prevalence of this process with an increase in salinity means similar microeukaryotic communities were selected for across the lakes, which is consistent with our previous hypothesis (climate change might select for specific traits) (Figure 4). In addition, we also found the dominance (freshwater lakes) and deficiency (salt lakes and brackish lakes) of dispersal limitation in the different lakes. Lower homogenizing dispersal was observed in the freshwater lakes than in the salt and brackish lakes, indicating that the probability of active dispersal in freshwater lakes was lower than that in other lakes (Nemergut et al., 2013).

## Data Availability Statement

The datasets presented in this study can be found in online repositories. The names of the repository/repositories and accession number(s) can be found below: https://www.ncbi.nlm.nih.gov/, PRJNA755855.

## Author Contributions

CL, GG, and BQ contributed the central idea of the manuscript and designed the experiments. XJ, KS, and XT performed the sample collection. FW and YH performed the experiment. CL analyzed most of the data and wrote the initial draft of the manuscript. FW carried out additional analyses and finalized this manuscript. All authors have read and approved the manuscript.

## Conflict of Interest

The authors declare that the research was conducted in the absence of any commercial or financial relationships that could be construed as a potential conflict of interest.

## Publisher’s Note

All claims expressed in this article are solely those of the authors and do not necessarily represent those of their affiliated organizations, or those of the publisher, the editors and the reviewers. Any product that may be evaluated in this article, or claim that may be made by its manufacturer, is not guaranteed or endorsed by the publisher.

## Footnotes

1
https://www.r-project.org
