# Supplementary material for: Salinity Is a Key Determinant for the Microeukaryotic Community in Lake Ecosystems of the Inner Mongolia Plateau, China
Source: Front Microbiol. 2022 Apr 12;13:841686. doi: 10.3389/fmicb.2022.841686 (PMC9039746; doi:10.3389/fmicb.2022.841686)
Supplement: Supplementary file 1 [file Data_Sheet_1.DOCX]

***Supplementary Material***

1. **Supplementary Tables and Figures**
   1. **Supplementary Tables**

Supplementary Table 1. Characteristics of the lakes in this study

| Lake | Sampling numbers | Elevation (m) | Salinity Level | Trophic Status | Water Surface Area (km^2^) | Mean water depth (m) |
| --- | --- | --- | --- | --- | --- | --- |
| Lake Daihai | 12 | 1221 | salt | eutrophic | 58.1 | 7.0 |
| Lake Dalinuoer | 13 | 1226 | brackish | eutrophic | 190.0 | 7.5 |
| Lake Durenaoer | 3 | 1276 | freshwater | eutrophic | 1.9 | 3.5 |
| Lake Chagannaoer | 3 | 1013 | freshwater | eutrophic | 30.2 | 3.0 |
| Lake Ganggengnuoer | 3 | 1243 | freshwater | eutrophic | 22.1 | 2.5 |

Supplementary Table 2. Taxonomic composition of the microeukaryotic community in lakes on the Inner Mongolia Plateau

|  | Sequences | % of sequences | OTUs | % of OTUs |
| --- | --- | --- | --- | --- |
| **Algae** | **601050** | **58.35%** | **570** | **22.12%** |
| Chlorophyta | 318267 | 30.90% | 192 | 7.45% |
| Dinophyceae | 121087 | 11.75% | 49 | 1.90% |
| Bacillariophyta | 96111 | 9.33% | 102 | 3.96% |
| Chrysophyceae | 44388 | 4.31% | 120 | 4.66% |
| Cryptophyceae | 10741 | 1.04% | 28 | 1.09% |
| Dictyochophyceae | 2919 | 0.28% | 9 | 0.35% |
| Synurophyceae | 2680 | 0.26% | 18 | 0.70% |
| Ochrophyta | 1869 | 0.18% | 26 | 1.01% |
| Eustigmatophyceae | 1789 | 0.17% | 8 | 0.31% |
| Haptophyta | 962 | 0.09% | 8 | 0.31% |
| Xanthophyceae | 146 | 0.01% | 8 | 0.31% |
| Chromeraceae | 67 | 0.01% | 1 | 0.04% |
| Bolidophyceae | 24 | 0.00% | 1 | 0.04% |
| **Protozoa** | **283864** | **27.56%** | **1113** | **43.19%** |
| Ciliophora | 91358 | 8.87% | 256 | 9.93% |
| Perkinsozoa | 87181 | 8.46% | 81 | 3.14% |
| Endomyxa | 38203 | 3.71% | 138 | 5.36% |
| Cercozoa | 26995 | 2.62% | 246 | 9.55% |
| Bigyra | 8187 | 0.79% | 144 | 5.59% |
| Choanozoa | 7317 | 0.71% | 42 | 1.63% |
| Imbricatea | 1388 | 0.13% | 23 | 0.89% |
| Apicomplexa | 426 | 0.04% | 21 | 0.81% |
| Haptista | 391 | 0.04% | 13 | 0.50% |
| Tubulinea | 209 | 0.02% | 11 | 0.43% |
| Evosea | 53 | 0.01% | 2 | 0.08% |
| Breviatea | 30 | 0.00% | 6 | 0.23% |
| Euglenozoa | 6 | 0.00% | 1 | 0.04% |
| Discosea | 3 | 0.00% | 1 | 0.04% |
| Other | 22117 | 2.15% | 128 | 4.97% |
| **Fungi** | **130166** | **12.64%** | **686** | **26.62%** |
| Cryptomycota | 50499 | 4.90% | 178 | 6.91% |
| Chytridiomycota | 34151 | 3.32% | 136 | 5.28% |
| Oomycota | 14987 | 1.45% | 61 | 2.37% |
| Ascomycota | 12391 | 1.20% | 40 | 1.55% |
| Basidiomycota | 1304 | 0.13% | 32 | 1.24% |
| Blastocladiomycota | 343 | 0.03% | 8 | 0.31% |
| Microsporidia | 32 | 0.00% | 3 | 0.12% |
| Hyphochytriomycetes | 18 | 0.00% | 3 | 0.12% |
| Zoopagomycota | 14 | 0.00% | 6 | 0.23% |
| Unclassified | 16427 | 1.59% | 219 | 8.50% |
| **Unclassified Eukaryota** | **15020** | **1.46%** | **208** | **8.07%** |
| **Total** | **1030100** | **100.00%** | **2577** | **100.00%** |

Supplementary Table 3. Partial Mantel test results showing comparisons between microbial community dissimilarity and a one-distance matrix, while controlling for the other two distance matrices

| Parameter | Salinity.dist controlling for Env.dist (excluding salinity)+ Geo.dist | Env.dist (excluding salinity) controlling for Geo.dist + salinity.dist | Geo.dist controlling for Env.dist (excluding salinity) + salinity.dist |
| --- | --- | --- | --- |
| r | 0.4702 | 0.5953 | 0.2968 |
| P | ＜0.001 | ＜0.001 | ＜0.001 |

Supplementary Table 4. Topological properties of the relevant random network

| Network indices | Salt lake | sd | Brackish lake | sd | Freshwater lakes | sd |
| --- | --- | --- | --- | --- | --- | --- |
| Average clustering coefficient | 0.07101 | 0.00993 | 0.07725 | 0.00638 | 0.07790 | 0.00153 |
| Average path distance | 2.59383 | 0.01619 | 2.38479 | 0.00672 | 2.06473 | 0.00184 |
| Modularity | 0.32957 | 0.01165 | 0.27157 | 0.00838 | 0.16140 | 0.00356 |
| Connectance | 0.07167 | 0 | 0.07759 | 0 | 0.07794 | 0 |
| Centralization.betweenness | 0.04760 | 0.01164 | 0.02741 | 0.00640 | 0.00540 | 0.00098 |
| Centralization.degree | 0.07364 | 0.01452 | 0.06893 | 0.01250 | 0.04719 | 0.00680 |

## Supplementary Figures


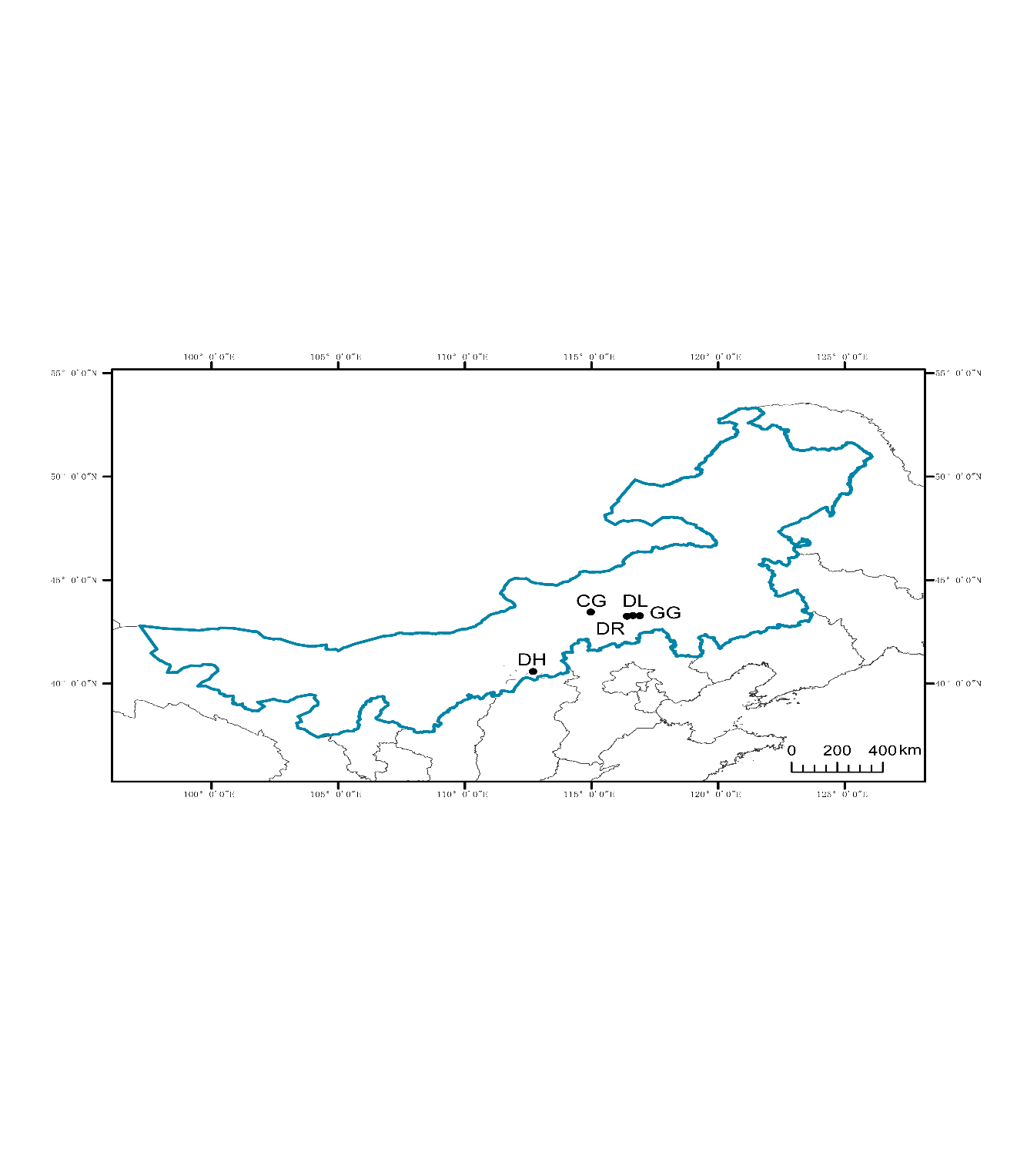


Supplementary Figure 1. Locations of the sampling sites in the Inner Mongolia Plateau. DH, DL, DR, CG and GG represent Lake Daihai, Lake Dalinuoer, Lake Durenaoer, Lake Chagannaoer and Lake Ganggengnuoer, respectively.


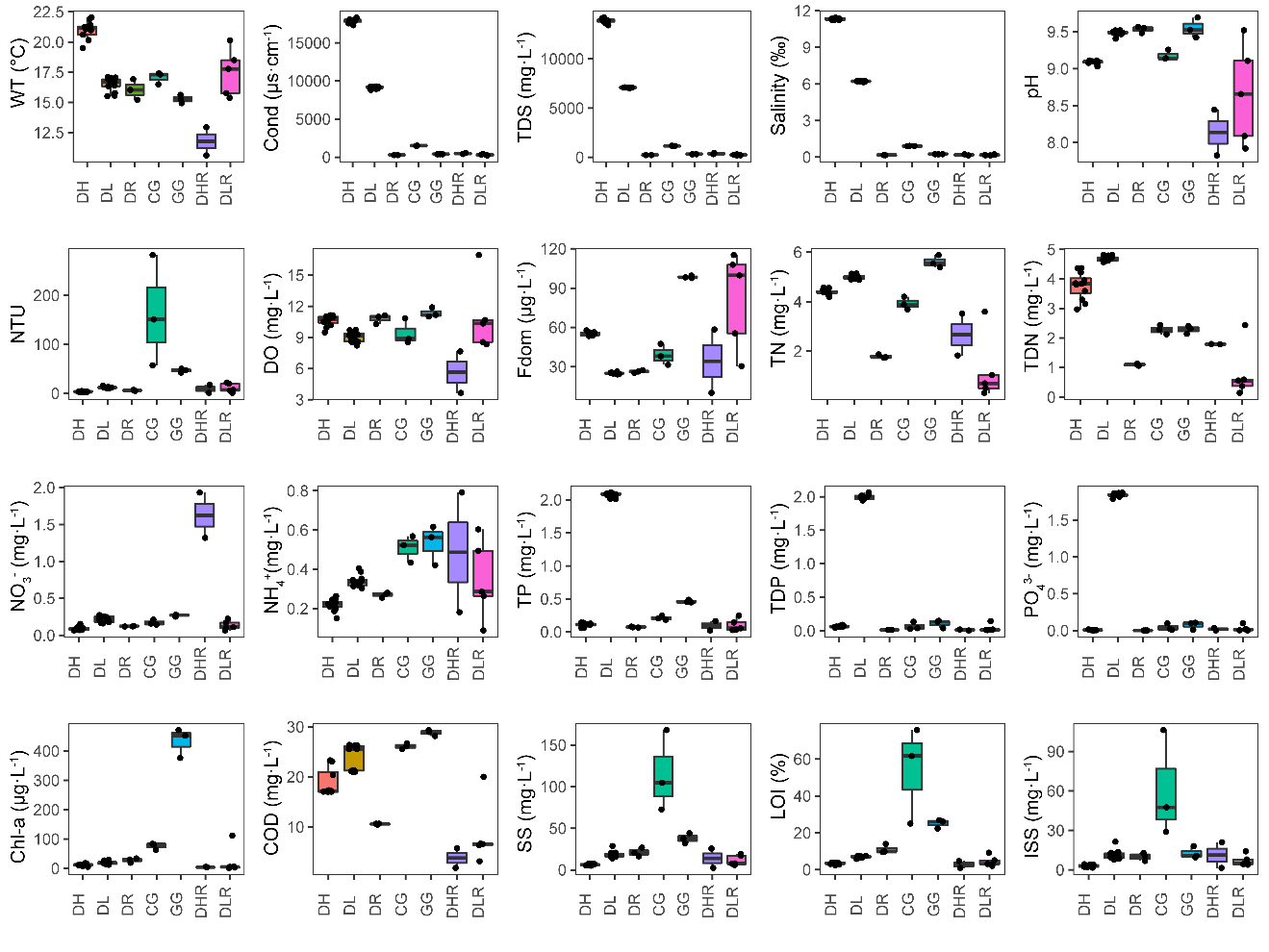


Supplementary Figure 2. Physicochemical properties of water samples from lakes. DH, DL, DR, CG and GG represent Lake Daihai, Lake Dalinuoer, Lake Durenaoer, Lake Chagannaoer and Lake Ganggengnuoer, respectively. DHR and DLR represent the inflow rivers of Lake Daihai and Lake Dalinuoer, respectively.


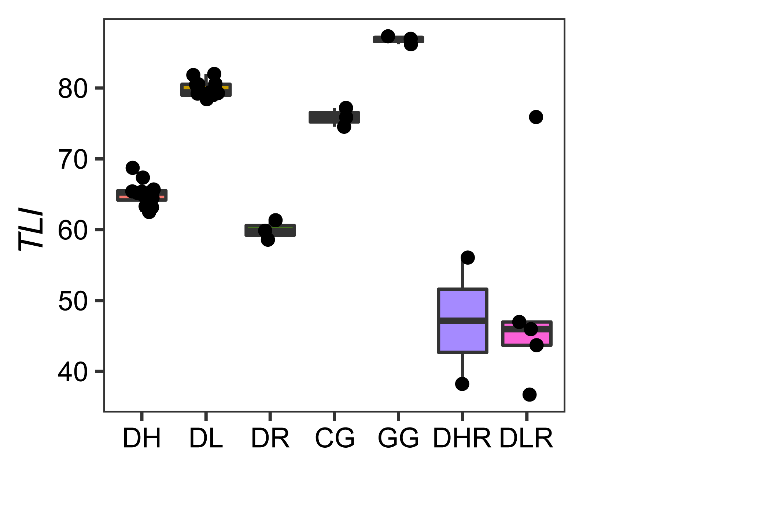


Supplementary Figure 3. The *TLI* among the lakes of the Inner Mongolia Plateau. DH, DL, DR, CG and GG represent Lake Daihai, Lake Dalinuoer, Lake Durenaoer, Lake Chagannaoer and Lake Ganggengnuoer, respectively. DHR and DLR represent the inflow rivers of Lake Daihai and Lake Dalinuoer, respectively.


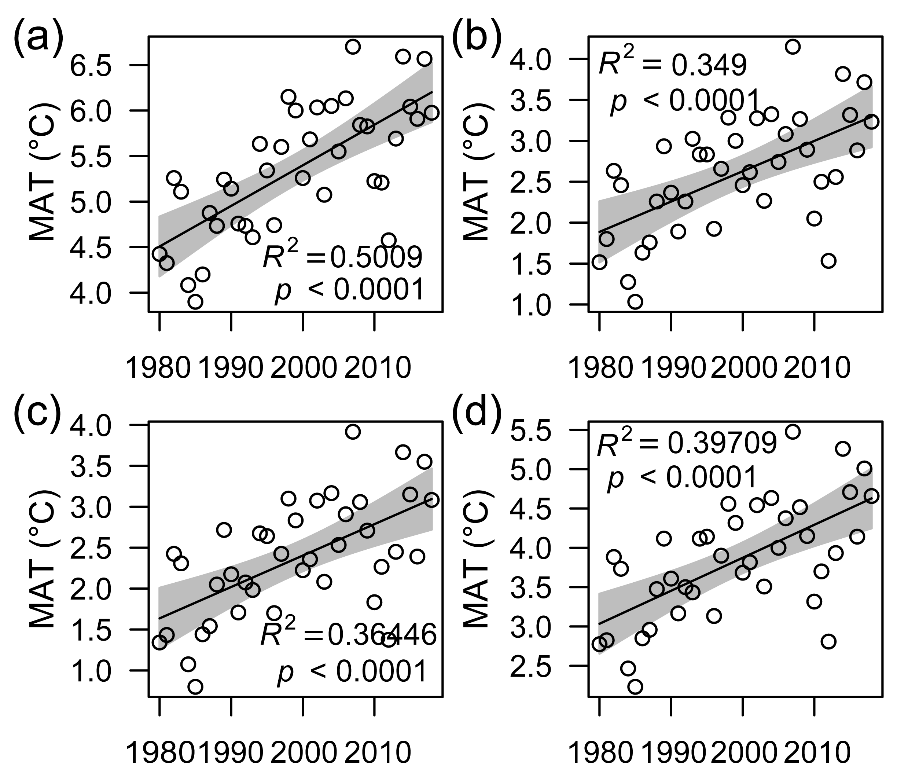


Supplementary Figure 4. The MAT among the lakes from 1980 to 2018. (a), (c) and (d) represent Lake Daihai, Lake Durenaoer, and Lake Chagannaoer, respectively. (b) Represents Lake Dalinuoer and Lake Ganggengnuoer.


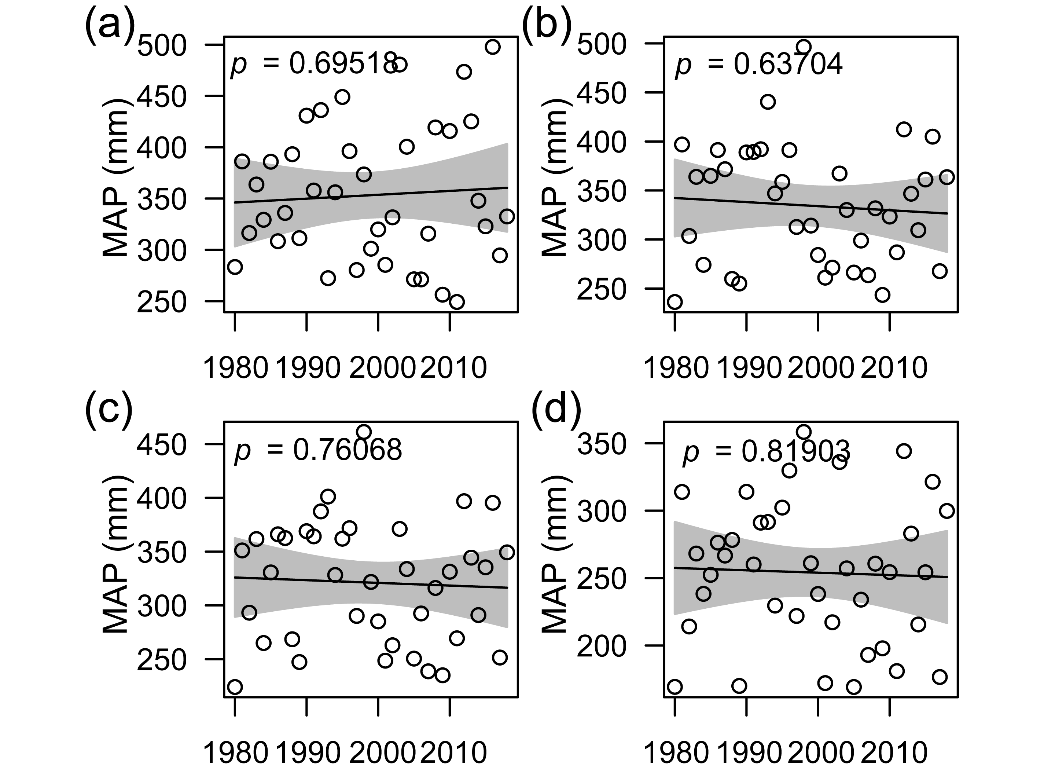


Supplementary Figure 5. The MAP among the lakes from 1980 to 2018. (a), (c) and (d) represent Lake Daihai, Lake Durenaoer, and Lake Chagannaoer, respectively. (b) Represents Lake Dalinuoer and Lake Ganggengnuoer.


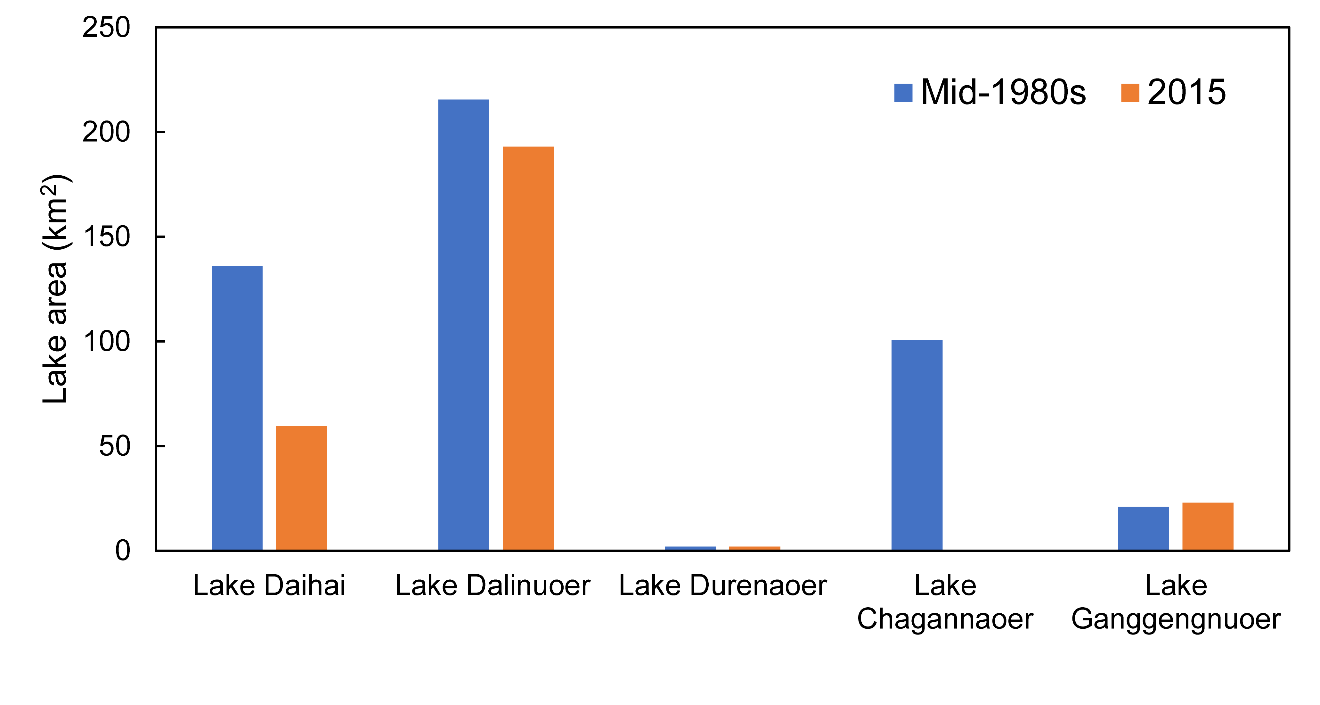


Supplementary Figure 6. The change in lake area from the mid-1980s to 2015 in Lake Daihai, Lake Dalinuoer, Lake Durenaoer, Lake Chagannaoer and Lake Ganggengnuoer.


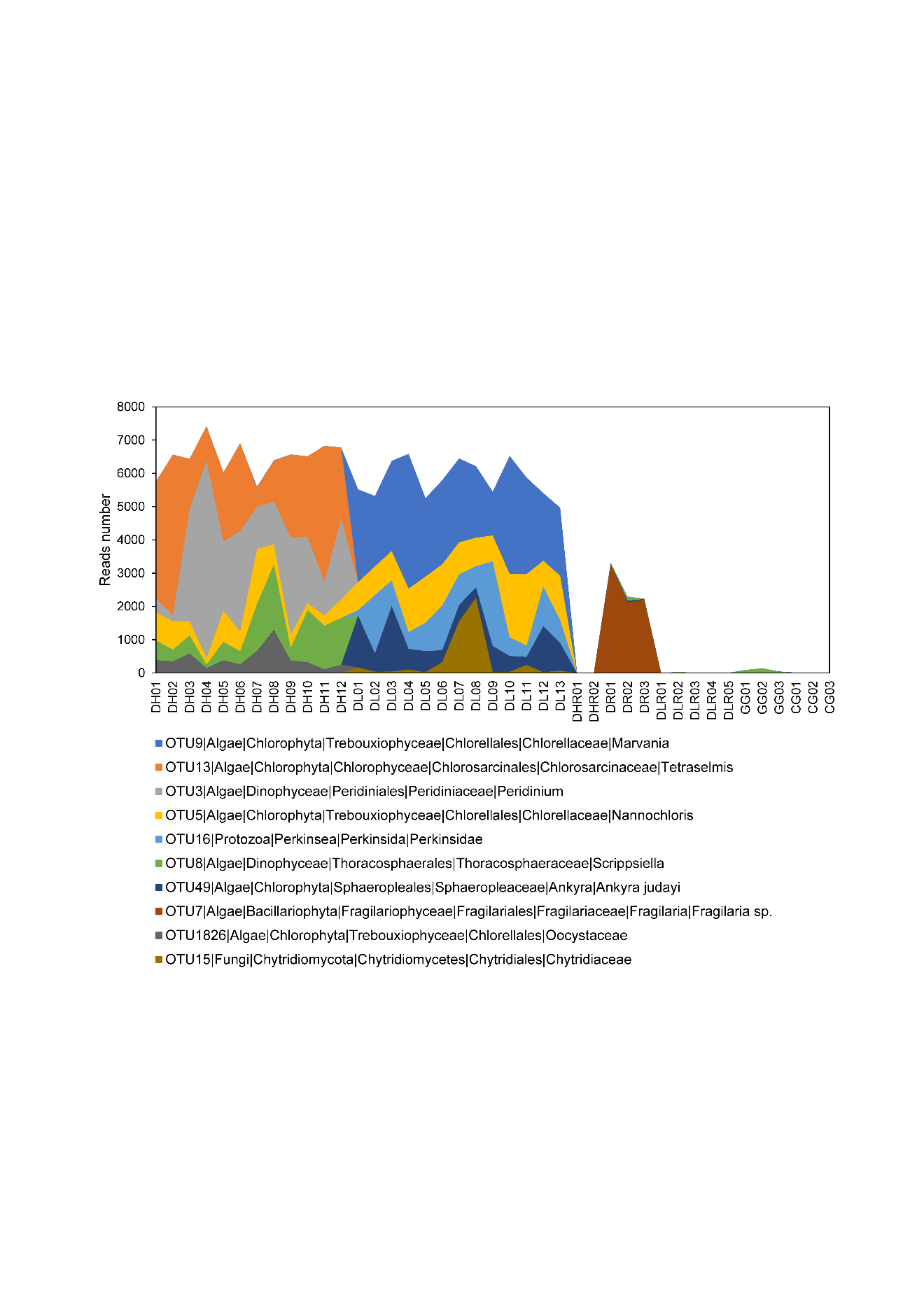


Supplementary Figure 7. The read numbers of the 10 most abundant OTUs among lakes with different salinity gradients on the Inner Mongolia Plateau. DH, DL, DR, CG and GG represent Lake Daihai, Lake Dalinuoer, Lake Durenaoer, Lake Chagannaoer and Lake Ganggengnuoer, respectively. DHR and DLR represent the inflow rivers of Lake Daihai and Lake Dalinuoer, respectively.


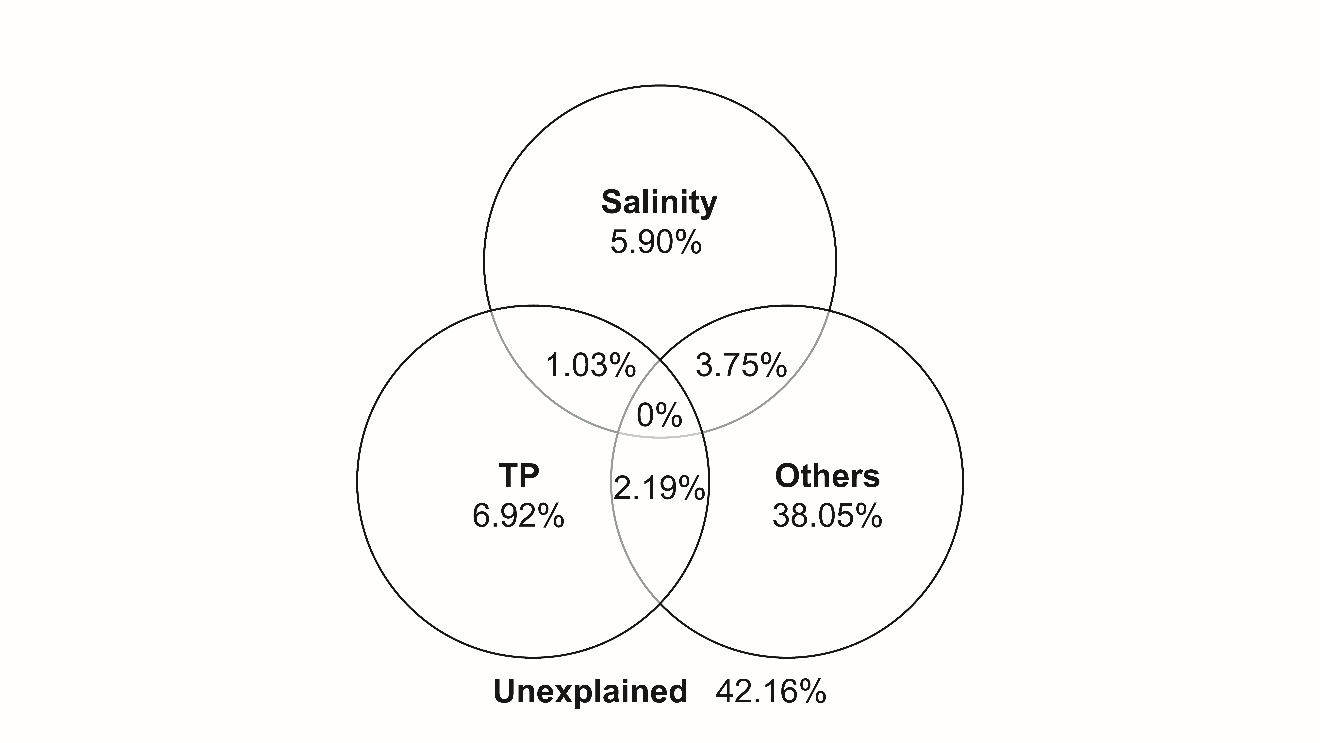


Supplementary Figure 8. VPA analysis result. Others includes Chl-a， NO_3_^-^， Fdom，NH_4_^+^，ISS and WT.


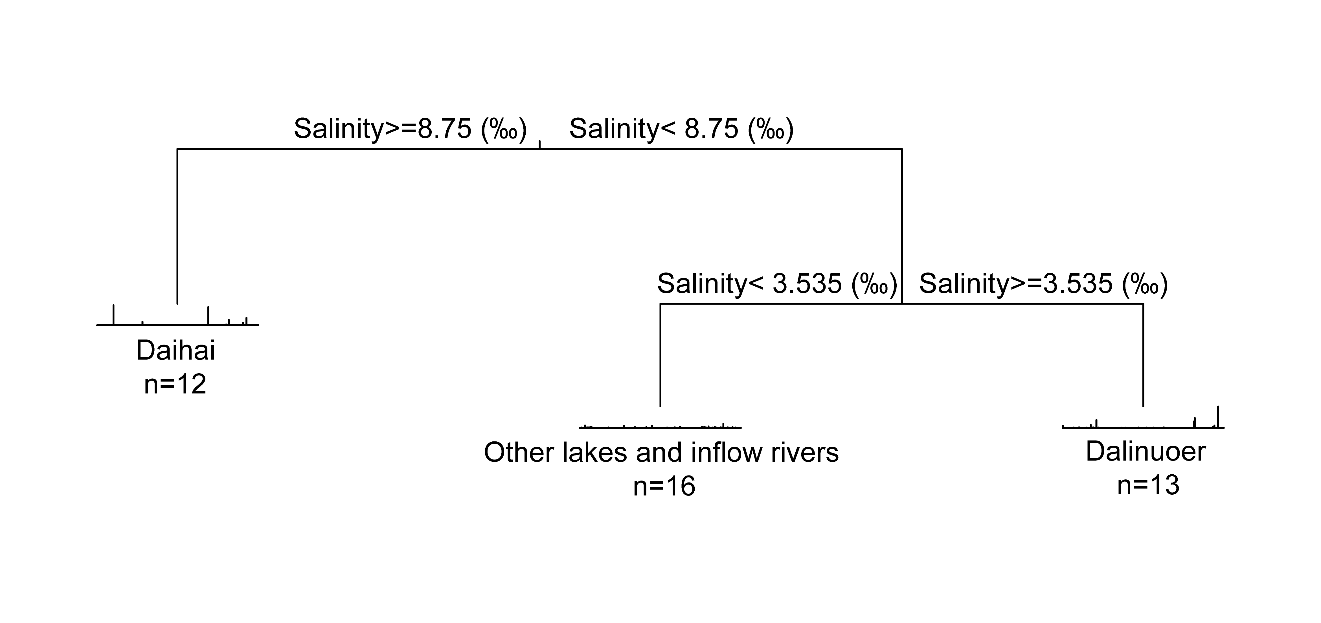


Supplementary Figure 9. Multivariate regression tree (MRT) analysis.


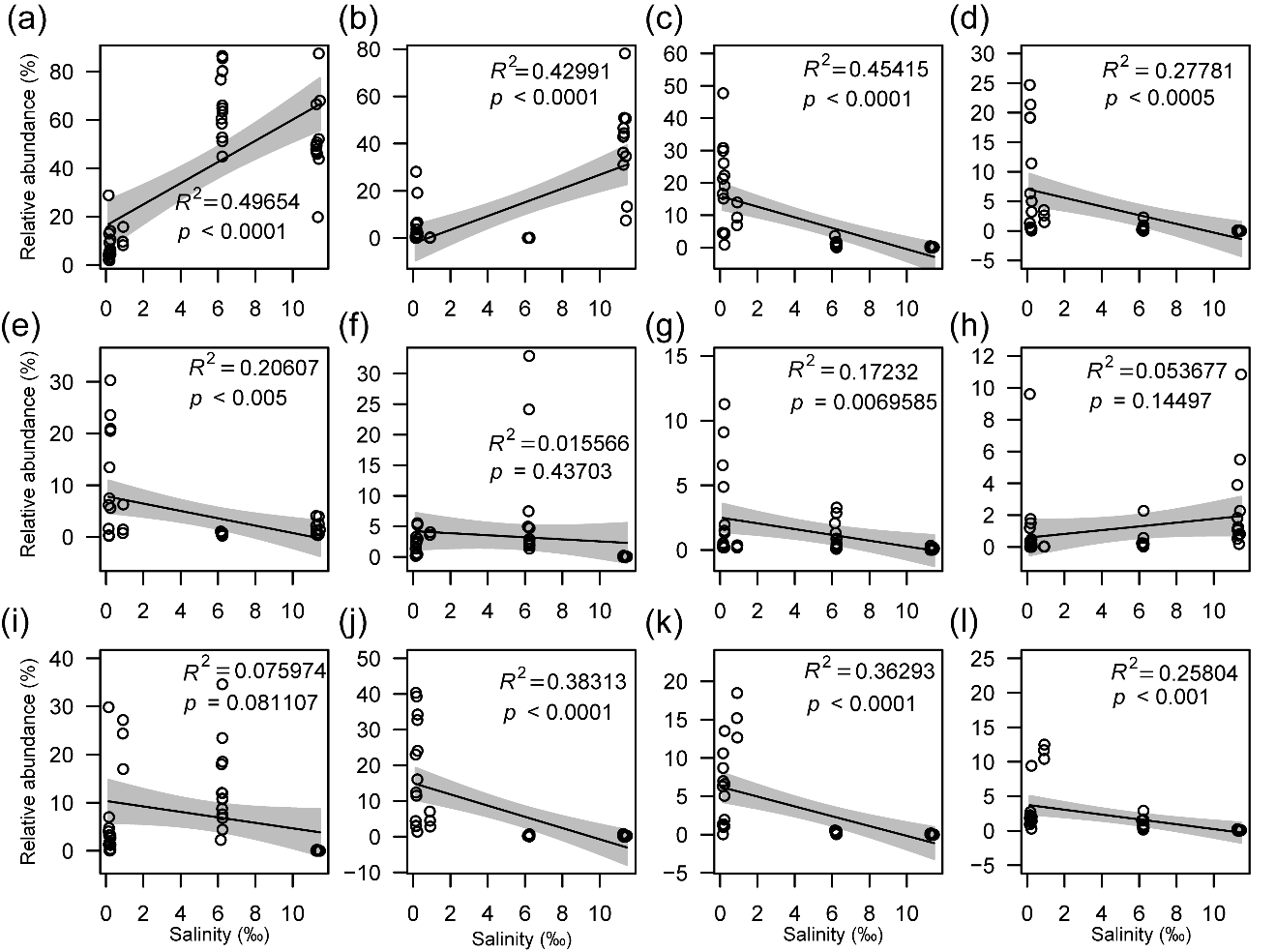


Supplementary Figure 10. The relative abundance of main microeukaryotic taxa at the phylum (or class) level. (a)-(l) represent Chlorophyta, Dinophyceae, Bacillariophyta, Chrysophyceae, Cryptomycota, Chytridiomycota, Oomycota, Ascomycota, Perkinsozoa, Ciliophora, Endomyxa and Cercozoa.


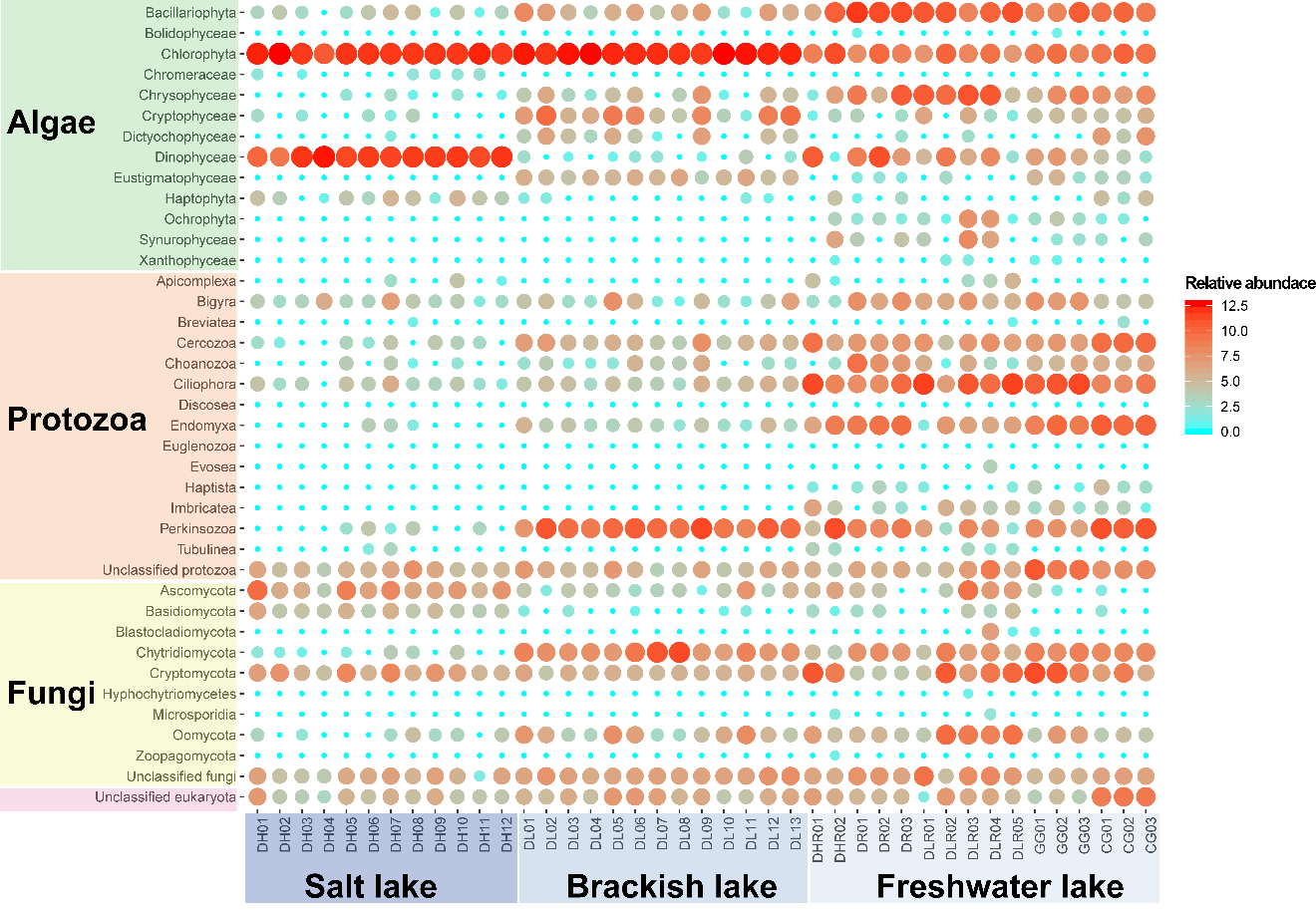


Supplementary Figure 11. Microeukaryotic community composition among lakes with different salinity gradients on the Inner Mongolia Plateau at the phylum (or class) level. DH, DL, DR, CG and GG represent Lake Daihai, Lake Dalinuoer, Lake Durenaoer, Lake Chagannaoer and Lake Ganggengnuoer, respectively. DHR and DLR represent the inflow rivers of Lake Daihai and Lake Dalinuoer, respectively.


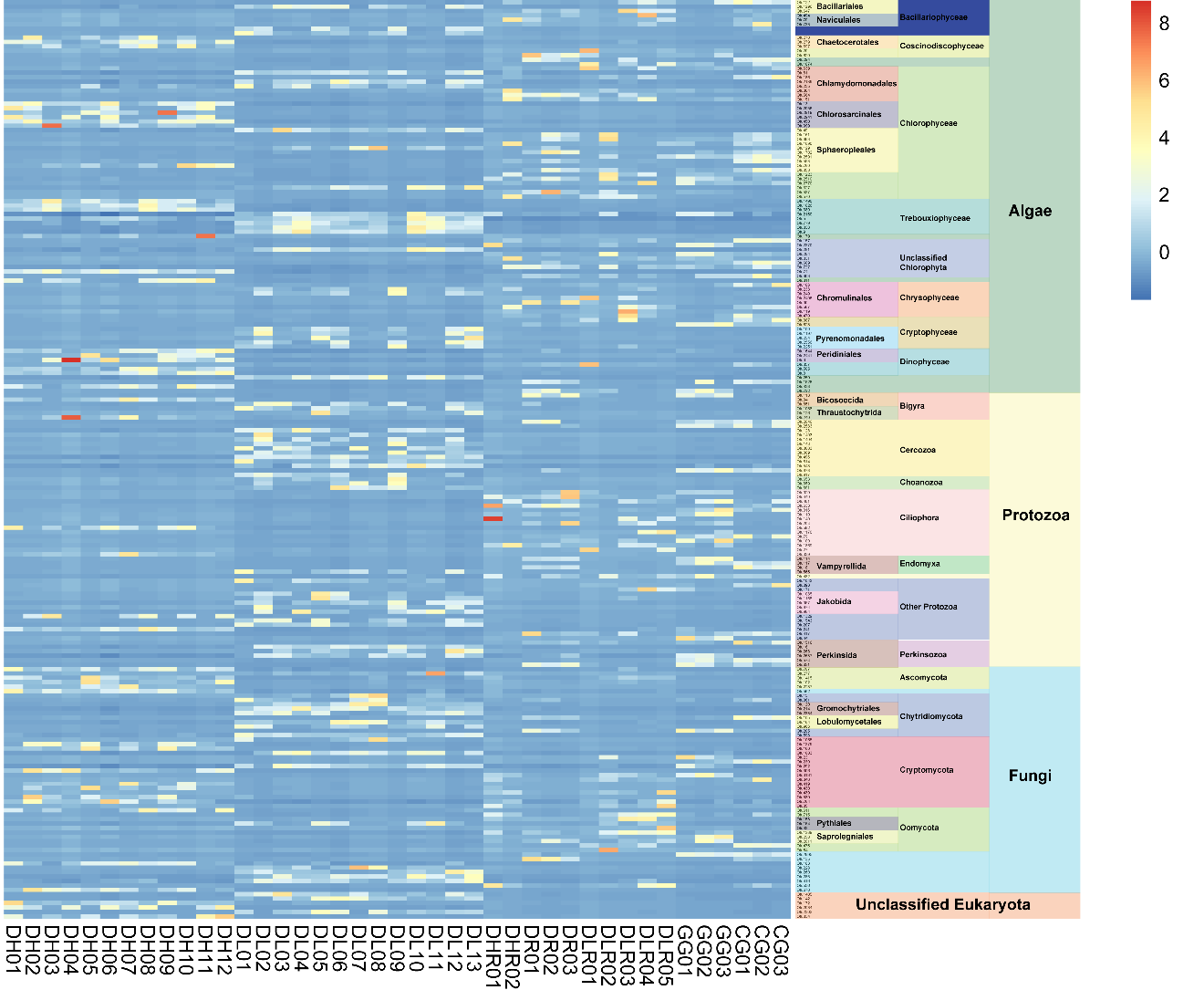


Supplementary Figure 12. Relative abundance distribution of indicator OTUs over the salinity gradient. DH, DL, DR, CG and GG represent Lake Daihai, Lake Dalinuoer, Lake Durenaoer, Lake Chagannaoer and Lake Ganggengnuoer, respectively. DHR and DLR represent the inflow rivers of Lake Daihai and Lake Dalinuoer, respectively.


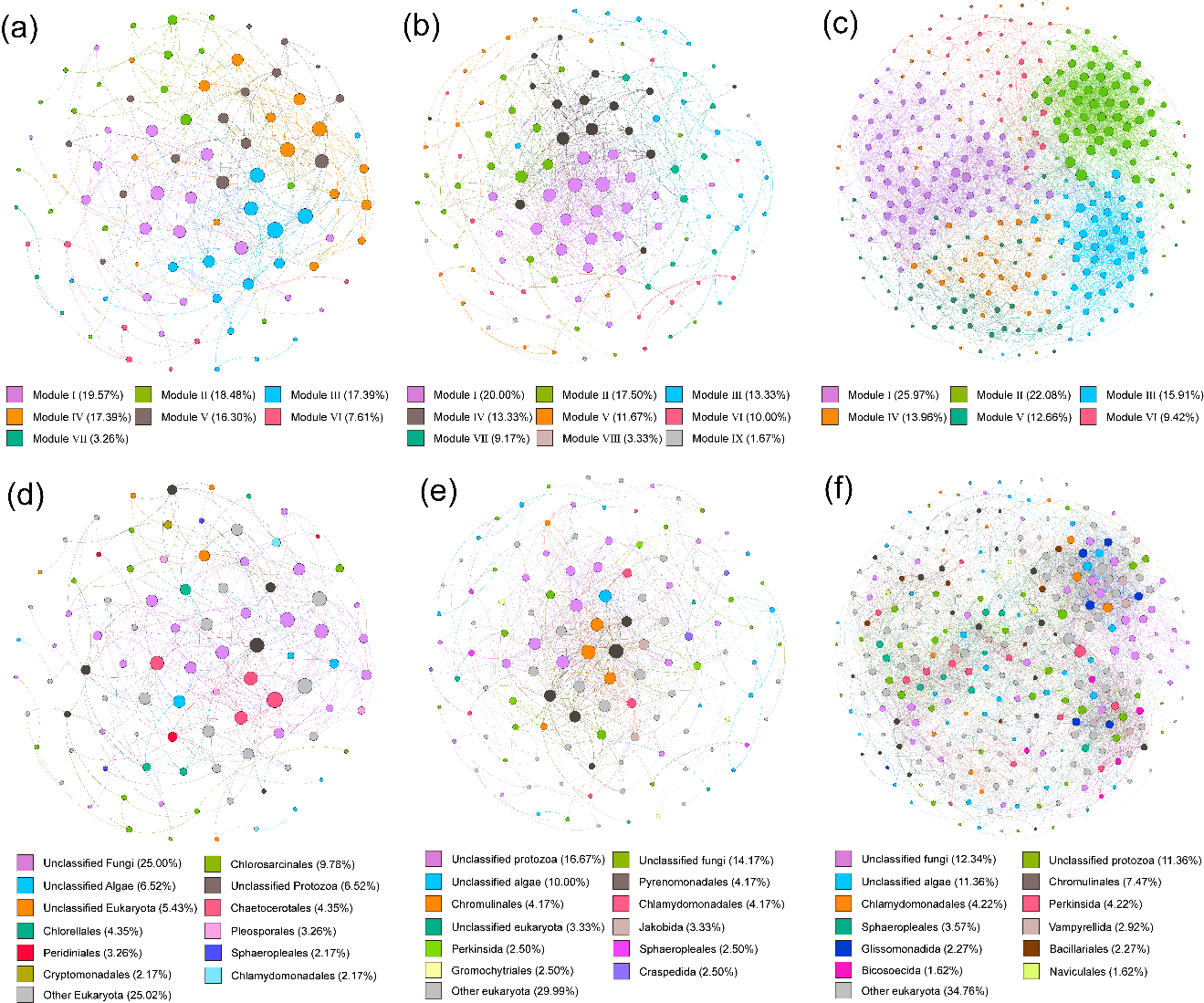


Supplementary Figure 13. Co-occurrence networks of microeukaryotic communities in lake systems on the Inner Mongolia Plateau. (a) and (d) represent salt lakes, (b) and (e) represent brackish lakes, and (c) and (f) represent freshwater lakes. Nodes of (a), (b) and (c) were colored according to different modularity classes, and (d), (e) and (f) were colored according to order level taxonomy. The size of the circles represents the degree of the node.


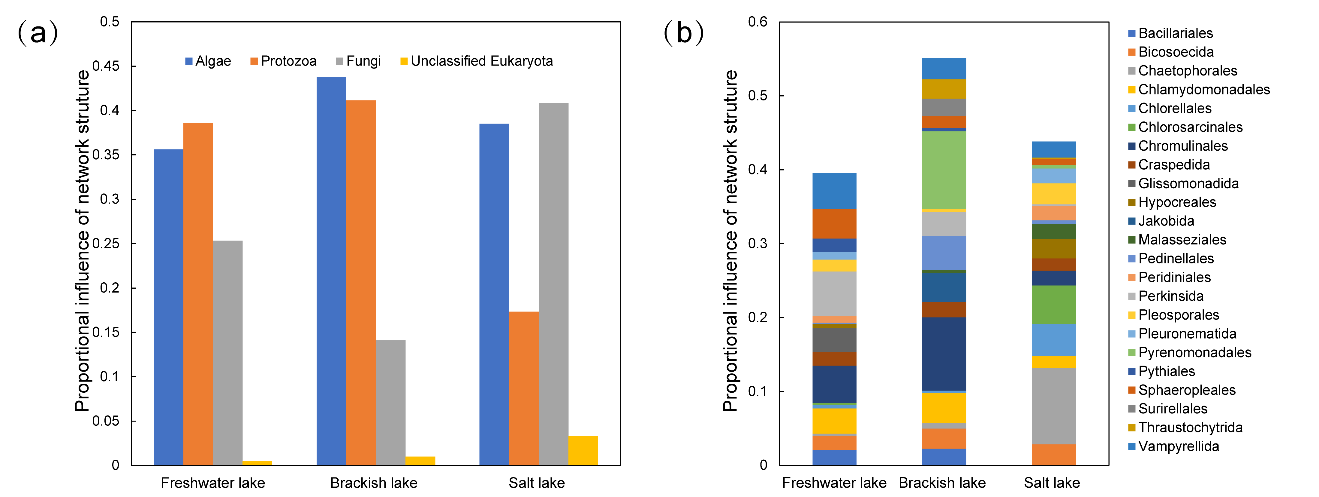


Supplementary Figure 14. The proportional influence of major (a) group and (b) order on the complexity of microeukaryotes among lakes with different salinity gradients on the Inner Mongolia Plateau.


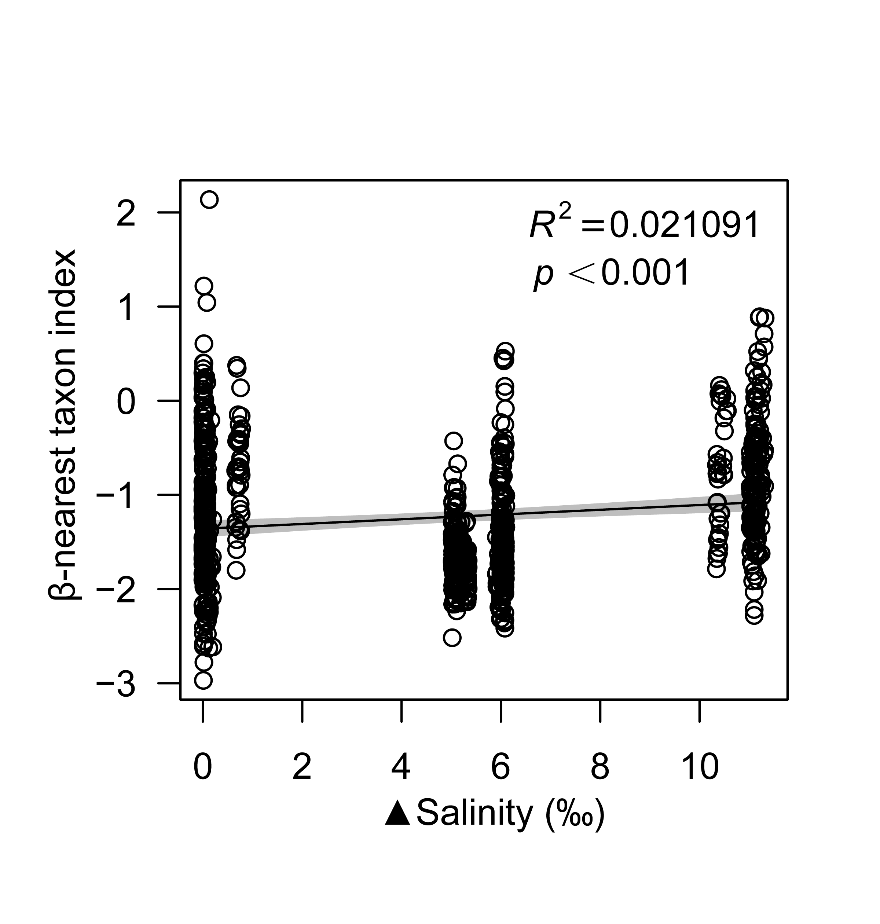


Supplementary Figure 15. Relationship between differences in salinity and *β*NTI.


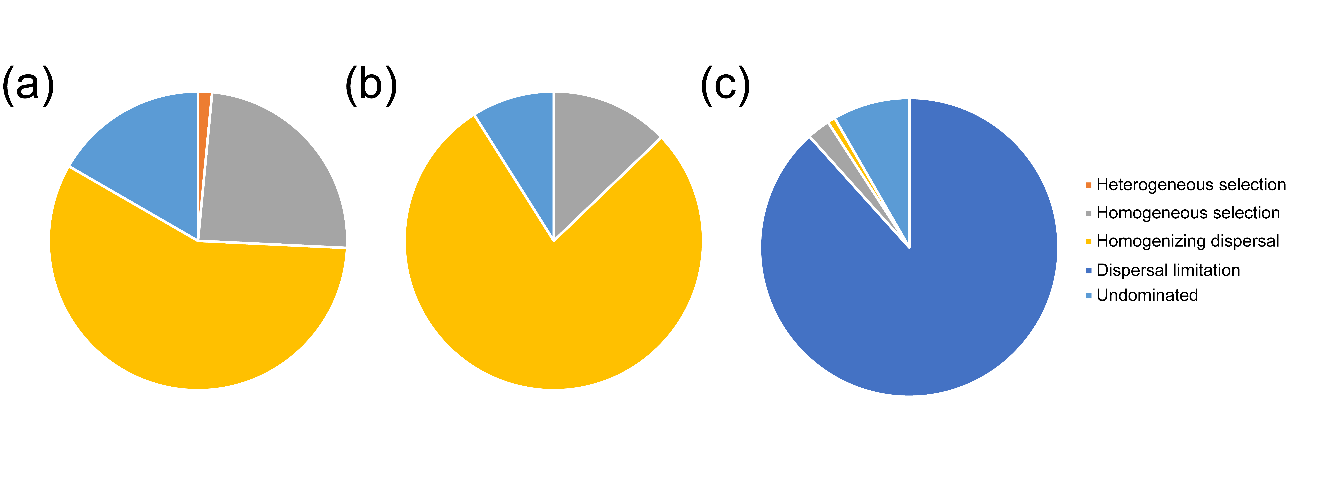


Supplementary Figure 16. Relative importance of ecological processes that govern the assembly of microeukaryotic communities among lakes with different salinity gradients on the Inner Mongolia Plateau. (a), (b) and (c) represent salt, brackish and freshwater lakes, respectively.
